# Mevalonate kinase-deficient THP-1 cells show a disease-characteristic pro-inflammatory phenotype

**DOI:** 10.3389/fimmu.2024.1379220

**Published:** 2024-03-14

**Authors:** Frouwkje A. Politiek, Marjolein Turkenburg, Rob Ofman, Hans R. Waterham

**Affiliations:** ^1^ Laboratory Genetic Metabolic Diseases, Department of Laboratory Medicine, Amsterdam University Medical Centers, Location Academic Medical Center, Amsterdam, Netherlands; ^2^ Amsterdam Gastroenterology Endocrinology Metabolism, Amsterdam, Netherlands; ^3^ Amsterdam Reproduction & Development, Amsterdam, Netherlands

**Keywords:** mevalonate kinase deficiency, autoinflammatory disorders, hyper IgD syndrome, innate immune response, cytokines, isoprenoid biosynthesis

## Abstract

**Objective:**

Bi-allelic pathogenic variants in the *MVK* gene, which encodes mevalonate kinase (MK), an essential enzyme in isoprenoid biosynthesis, cause the autoinflammatory metabolic disorder mevalonate kinase deficiency (MKD). We generated and characterized MK-deficient monocytic THP-1 cells to identify molecular and cellular mechanisms that contribute to the pro-inflammatory phenotype of MKD.

**Methods:**

Using CRISPR/Cas9 genome editing, we generated THP-1 cells with different MK deficiencies mimicking the severe (MKD-MA) and mild end (MKD-HIDS) of the MKD disease spectrum. Following confirmation of previously established disease-specific biochemical hallmarks, we studied the consequences of the different MK deficiencies on LPS-stimulated cytokine release, glycolysis versus oxidative phosphorylation rates, cellular chemotaxis and protein kinase activity.

**Results:**

Similar to MKD patients’ cells, MK deficiency in the THP-1 cells caused a pro-inflammatory phenotype with a severity correlating with the residual MK protein levels. In the MKD-MA THP-1 cells, MK protein levels were barely detectable, which affected protein prenylation and was accompanied by a profound pro-inflammatory phenotype. This included a markedly increased LPS-stimulated release of pro-inflammatory cytokines and a metabolic switch from oxidative phosphorylation towards glycolysis. We also observed increased activity of protein kinases that are involved in cell migration and proliferation, and in innate and adaptive immune responses. The MKD-HIDS THP-1 cells had approximately 20% residual MK activity and showed a milder phenotype, which manifested mainly upon LPS stimulation or exposure to elevated temperatures.

**Conclusion:**

MK-deficient THP-1 cells show the biochemical and pro-inflammatory phenotype of MKD and are a good model to study underlying disease mechanisms and therapeutic options of this autoinflammatory disorder.

## Introduction

Mevalonate kinase (MK) is an essential enzyme of the isoprenoid biosynthesis pathway. This metabolic pathway produces sterol and non-sterol isoprenoids that play pivotal roles in multiple cellular processes and the regulation thereof. The importance of this pathway in the regulation of the innate immune response became clear when biallelic loss-of-function variants in the *MVK* gene, encoding MK, were found to be the cause of the autoinflammatory disease Hyper IgD syndrome (MKD-HIDS, MIM# 260920) (1, 2). MKD-HIDS is characterized by a lifelong occurrence of episodic inflammation. These episodes generally last 3 to 7 days and can be accompanied with various symptoms, including high fever, malaise, headache, abdominal pain, nausea, lymphadenopathy, joint pain, skin rash, splenomegaly and hepatomegaly. In most patients the first inflammatory episode is triggered by childhood vaccinations. Apart from vaccinations, infections and stress can also induce inflammatory episodes. However, the episodes often appear to occur without a clear cause (3–5). This recurrent, unprovoked occurrence of inflammatory episodes is characteristic for the increasing group of systemic autoinflammatory diseases (SAIDs (6);).

Before the identification of MK deficiency as the underlying defect of MKD-HIDS, MK deficiency had already been identified as the cause of the rare, severe inborn error of metabolism mevalonic aciduria (MKD-MA, MIM# 610377) (7). Patients with MKD-MA often present with rather severe clinical features, including dysmorphic features, failure to thrive, psychomotor delay, ocular involvement, hypotonia, progressive cerebellar ataxia, and myopathy. In some patients, MKD-MA results in death in early childhood (8, 9). Interestingly, however, similar to MKD-HIDS, patients with MKD-MA also experience the recurrent inflammatory episodes. Nowadays, mevalonate kinase deficiency (MKD) is considered a disease spectrum that ranges from the milder affected MKD-HIDS patients to the severely affected MKD-MA patients (10).

For most MKD patients, the severity of clinical symptoms correlates well with the residual MK activities and protein levels in cells, i.e. peripheral blood mononuclear cells (PBMCs) or primary skin fibroblasts. The residual MK activities in cells from MKD-MA patients are typically less than 1% of the activities in cells from healthy individuals and often below the detection limit (9, 11), while MK activities in cells from MKD-HIDS patients usually range from 2 to 10% of the activities in cells from healthy individuals (1, 2, 12–14). The differences in residual MK activities are also reflected in the urinary levels of mevalonic acid, the substrate of MK. In MKD-MA there often is a persistent urinary excretion of high levels of mevalonic acid, while in MKD-HIDS urinary mevalonic acid levels are only moderately elevated during inflammatory episodes (1, 2, 9).

Similar as in many other monogenic SAIDs, the inflammation in MKD is driven by the pro-inflammatory cytokine interleukin-1β (IL-1β) (15). PBMCs from MKD patients already secrete increased basal amounts of IL-1β as well as other pro-inflammatory cytokines (14, 16–18), and this secretion becomes massive upon lipopolysaccharides (LPS) stimulation. This enhanced secretion was shown to be a consequence of a shortage of non-sterol isoprenoids (14, 17), in particular geranylgeranyl-PP, which is required for the posttranslational prenylation of proteins (19). Accordingly, several studies showed that the prenylation of the small Rho and Rab GTPases is compromised in cells from MKD-MA patients or becomes compromised in cells from MKD-HIDS patients at elevated temperatures, which cause a further decrease of the residual MK activities (20–24).

Although the involvement of the isoprenoid biosynthesis pathway, including a (temporary) compromised protein prenylation, has been established, it is currently still not fully understood how, when and why the inflammatory episodes occur in MKD. Many of the currently known biochemical and cellular consequences in MKD have been studied first in primary skin fibroblasts of patients and were subsequently confirmed in patient PBMCs. Because skin fibroblasts do not allow studies on inflammation and the (dys)regulation of the innate response, and patient PBMCs are not easy to obtain and/or to manipulate, we used the CRISPR/Cas9 genome editing technology to generate severe (MKD-MA) and mild (MKD-HIDS) MK deficiency in the human THP-1 monocyte cell line. After genetic and biochemical validation of the different cell lines, we studied the consequences of the different MK-deficiencies on protein prenylation (Rap1a geranylgeranylation), the pro-inflammatory response (LPS-induced cytokine release), cellular metabolism (oxidative phosphorylation versus glycolysis rates), cell migration (chemotaxis experiments) and protein kinase activity (kinome analysis).

## Methods

### Cell culture

THP-1 cells were cultured at 37°C in a humidified atmosphere containing 5% CO_2_ in RPMI 1640 medium (Gibco) containing 3.6 mM glutamine (Gibco), 90 U/ml penicillin and streptomycin (Gibco), 5.0 mg/l plasmocin (Invivogen) and 10% heat-inactivated (30 minutes at 56°C) fetal bovine serum (Capricorn Scientific, FBS-12A).

### Materials

Geranylgeraniol (GGOH; #G3278), lapaquistat acetate (TAK-475; #SML2168), simvastatin (#S6196), PP2 (#HY-13805) and dasatinib (#CDS023389) were purchased from Sigma, SU-6656 (sc-203286) was purchased from Santa Cruz.

### Generation of MK-deficient THP-1 cells by CRISPR/Cas9 genome editing

The MKD-MA THP-1 cell line used in this study was generated by introducing deletions in exon 11 of the *MVK* gene using the CRISPR/Cas9 genome editing protocol of Integrated DNA Technologies (IDT). To this end, we designed an Alt-R^®^ CRISPR-Cas9 crRNA, which contains a 20 nt sequence that is identical to the genomic sequence downstream of a proto-spacer adjacent motif (PAM) site in exon 11 (5′-CGG, c.1125_1127) of the *MVK* gene using the online Alt-R Custom Cas9 crRNA Design tool (IDT). There were no significant putative off-target regions predicted by the online design tool (IDT) for this crRNA. Alt-R^®^ CRISPR-Cas9 ATTO™ 550-labeled tracrRNA and the Alt-R^®^ CRISPR-Cas9 crRNA targeting *MVK* exon 11 were resuspended in nuclease-free IDTE (pH 7.5) to a final concentration of 100 µM, mixed in equimolar concentrations and then diluted with nuclease-free duplex buffer to a tracrRNA:crRNA duplex concentration of 25 µM. After heating at 95°C for 5 minutes, the tracrRNA:crRNA duplex was left to cool down to room temperature. Next, the ribonucleoprotein (RNP) complex was formed by adding Alt-R^®^ S.p. HiFi Cas9 Nuclease V3 to the tracrRNA:crRNA duplex in a 1:1.2 molar ratio, followed by 20 minutes incubation at room temperature. Transfections were performed using the Amaxa Cell Line Nucleofector Kit V and the Amaxa Nucleofector II (Lonza). In short, 1 x 10^6^ THP-1 cells were resuspended in 100 µl nucleofector solution and transfected with 6.4 µl of the RNP complex and 4 µl of Alt-R Cas9 Electroporation Enhancer (stock 100 µM in nuclease-free IDTE, pH 7.5) using program V-01. Directly following transfection, medium was added to the cells, and the cells were transferred to a 24-wells plate with a final volume of 1.5 ml standard culture medium per well. After 24 hours, cells were seeded by FACS sorting (SH800 Sony sorter) one cell per well into 96-well plates, containing 15% ‘THP-1-conditioned medium’. THP-1-conditioned medium was obtained by culturing 2 x 10^5^ THP-1 cells/ml for 24 hours under standard culture conditions. The cells were then centrifuged, and the supernatant was frozen as THP-1-conditioned medium at -20°C. Before use, the THP-1-conditioned medium was passed through a 0.45 μm filter (Millipore Millex-HP) and diluted in standard culture medium. Surviving clonal cell lines were analyzed for MK protein levels by western blotting and the entire *MVK* gene was analyzed by Sanger sequencing. After screening >200 clonal cell lines we identified only one cell line that showed hardly any MK protein on western blots, undetectable MK enzyme activity and which appeared compound heterozygous for an out-of-frame deletion (c.1121_c.1130del (p.(Gly376Serfs*98))) and an in-frame deletion (c.1112_c.1129del (p.(Ser371_Val377delinsIle))) in exon 11.

To verify that metabolic, immunological and cellular changes in this MKD-MA cell line are caused by the introduced MK deficiency and not by an off-target event, we stably expressed wild-type (WT) *MVK* cDNA by transfecting the cells with 0.5 µg pcDNA5/FRT-*MVK* (Invitrogen) using the AMAXA Cell Line Nucleofector Kit V (Lonza). Three days after transfection, transfected cells were selected by culturing them in the presence of RPMI with 10% heat-inactivated delipidated FBS (Capricorn) for approximately 5 weeks (note: non-transfected MKD-MA cells appeared not viable on delipidated FBS). Following selection, the cells were cultured in standard culture medium. Using FACS sorting (SH800 Sony sorter) the selected cells were seeded one cell per well into 96-well plates, containing 15% THP-1-conditioned medium. Expression of WT MK protein in the selected clonal cell lines was confirmed by western blotting.

The generation of the two MKD-HIDS cell lines used in this study was performed by GENETAGUS (https://genetagus.com) using a similar strategy as described above. In short, a CRISPR/Cas9 knock-in strategy was used to introduce the common c.1129G>A (p.V377I) variant in the *MVK* gene in THP-1 cells. Only a few heterozygous cell clones were obtained, which were used for a second round. This resulted in two clonal cell lines homozygous for the p.V377I variant, both originating from the same heterozygous clone. MK protein levels were determined by western blotting. Sanger sequencing of the entire *MVK* gene was performed to verify the homozygous presence of the p.V377I variant and exclude the presence of additional variants.

### Biochemical assays

THP-1 cells were incubated in 12-well plates (1 x 10^6^ cells/ml, 1 ml/well) for 24 hours or 72 hours in standard culture medium. Pellets were made to measure MK enzyme activity as previously described (25) using ^14^C-labeled mevalonate. Mevalonic acid levels in culture medium were determined as previously described for urinary samples (26).

### Cytokine measurements

For cytokine measurements, THP-1 cells were incubated in 12-well plates (1 x 10^6^ cells/ml, 1 ml/well) with or without the indicated compounds. After 20 hours of culturing, lipopolysaccharides (LPS) from *Escherichia coli* EH100 (Alexis Biochemicals, Enzo life sciences) was added and the cells were cultured for an additional 4 hours (final LPS concentration 10 ng/ml). After centrifugation for 5 min, 1500 rpm at 4°C, supernatants were transferred to clean tubes and frozen at -20°C.

The levels of IL-1β, tumor necrosis factor alpha (TNF-α) and interleukin-6 (IL-6) were measured in the supernatants by enzyme-linked immunosorbent assays (ELISAs) (R&D systems). The ELISAs were performed according to the manufacturer’s instructions and all samples were measured in duplicate.

### Western blot analysis

Cell homogenates were prepared by sonication (40 J/Ws) in Tris-buffered saline (TBS) supplemented with Complete Mini protease inhibitors (Roche). For the detection of phosphorylated proteins, HALT phosphatase inhibitor (Thermo Fisher Scientific) was added to the cell homogenates. Protein concentrations were quantified using the BCA Protein Assay (Thermo Scientific). Lithium dodecyl sulfate (LDS) sample buffer and sample reducing agent (NuPage, Invitrogen) were added to the cell homogenates as described in the manufacturer’s protocol. After heating the samples for 10 min at 70°C, equal amounts of protein were separated using 10% Bis-Tris protein gels (NuPage, Invitrogen). To determine the size of the proteins, we used the Precision Plus Protein Dual Color Standards (Bio-Rad). Electrophoresis was performed in 3-(N-Morpholino) propanesulfonic acid (MOPS) running buffer, after which the proteins were transferred to a polyvinylidene difluoride (PVDF) membrane (for the primary antibody against unprenylated Rap1a) or to a nitrocellulose membrane (for all other primary antibodies) using the iBlot Dry Blotting System (iBlot 2 Gel Transfer Device). Membranes were blocked in 2% bovine serum albumin (BSA; Sigma) in TBS with 0.1% Tween-20 (TBS-T) for 1 hour at room temperature, followed by overnight incubation at 4°C with primary antibodies in 2% BSA in PBS-T. Primary antibodies were used against MK (1:5,000, own production (27), HMGCR (1:1,000, Atlas antibodies, AMAb90618), unprenylated Rap1a (1:500, Santa-Cruz, sc-373968), total Rap1 (1:500, Santa-Cruz, sc-398755), SRC (1:1,000, Cell Signaling, 2110) and phosphorylated Src family kinases (Tyr416) (1:1,000, Cell Signaling, 2101). As loading control, membranes were incubated with a primary antibody against α-tubulin (1:2,000, Sigma-Aldrich, T6199). For visualization, we used the secondary antibodies IRDye 800CW goat anti-rabbit, IRDye 800CW goat anti-mouse or IRDye 680RD donkey anti-mouse (LI-COR Biosciences) at a 1:10,000 dilution in a 1:1 dilution of TBS-T and InterceptTM (PBS) blocking buffer (LI-COR Biosciences) with 0.02% SDS. The membranes were incubated with secondary antibodies for 2 hours at room temperature. For visualization, the membranes were scanned and quantified using the Odyssey Infrared Imaging System and software (LI-COR Biosciences).

### Chemotaxis assay

The migration of THP-1 cells was studied using a 24-well transwell chemotaxis assay. Briefly, THP-1 cells were plated in 6-well plates (1 x 10^6^ cells/ml, 3 ml/well) and cultured for 24 hours in standard medium with or without 10 ng/ml LPS. Next day, 200 µl containing 1 x 10^6^ cells was added to 24-well inserts (Greiner Bio-One ThinCert™ CellCoat™, pore size 8 µM). The inserts were directly placed in a 24-wells plate containing 1 ml standard cultured medium with or without 25 ng/ml monocyte chemoattractant protein-1 (MCP-1, R&D systems). THP-1 cells were allowed to migrate for 1 hour at 37°C in a humidified atmosphere containing 5% CO_2._ The number of cells that migrated to the lower chamber was determined using the Muse™ Cell Analyser and the Muse™ Count & Viability Kit.

### Seahorse XF measurements

We used the Seahorse XF Glycolytic Rate Assay Kit and the Seahorse XFe 96 Analyzer (Agilent Technologies) to measure the real time extracellular acidification rate (ECAR; measure for glycolysis), and oxygen consumption rate (OCR; measure for mitochondrial oxidative phosphorylation) in the different THP-1 cells. The OCR and ECAR measurements were used to determine the glycolytic proton efflux rate (glycoPER), which is the total proton efflux rate corrected for the acidification caused by mitochondrial function (CO_2_ produced by the TCA cycle).

One day before the analysis, the sensor cartridge was hydrated in Seahorse XF Calibrant (Agilent Technologies) and incubated overnight at 37°C in a non-CO_2_ incubator. THP-1 cells were plated in a T25 cell culture flask (2 x 10^5^ cells/ml) and incubated for 24 hours without LPS, or for 4 or 24 hours with 10 ng/ml LPS. The cells were then centrifuged and resuspended in Seahorse XF DMEM medium, pH 7.4 (Agilent Technologies) supplemented with 10 mM glucose, 1 mM sodium pyruvate and 2 mM glutamine. Next, 1 x 10^5^ cells in 50 µl Seahorse XF DMEM medium per well were plated onto Cell-Tak (Corning) coated XF96 cell culture microplates (Agilent Technologies). The microplates were centrifuged for 1 minute at 200 x g (low acceleration and no braking) after which 130 µl Seahorse XF DMEM medium was added to each well. The plate was then placed in a non-CO_2_ incubator at 37°C for 1 hour. The analysis was performed in a Seahorse XFe 96 Analyzer according to the manufacturer’s instructions (Agilent Technologies), using a final concentration of 0.5 µM rotenone, 0.5 µM antimycin A and 50 mM 2-deoxy-d-glucose.

After the analysis, the Seahorse medium was discarded and the cells were lysed in 50 µl/well 0.1 M NaOH for protein quantification using the Bradford assay (Bio-Rad).

### Kinome analysis

Kinome analysis was performed using the PamGene functional kinase activity profiling technology. To this end, THP-1 WT and MKD-MA cells were cultured in T75 flasks (1 x 10^6^ cells/ml) for 24 hours, under standard culture conditions. Subsequently, cells were lysed in M-Per Mammalian Protein Extraction Reagent substituted with HALT protease inhibitor cocktail EDTA-free and HALT phosphatase inhibitor (Thermo Fisher Scientific). Lysates were prepared according to the manufacturer’s protocol (protocol 1160, PamGene), aliquoted and stored at –80°C until further use. Protein concentrations were determined using the Bradford assay (Bio-Rad). Protein Tyrosine Kinase (PTK) and Serine/Threonine Kinase (STK) peptide arrays (PamGene) were used to measure the activity of protein kinases in the cell lysates. In short, the PTK and STK peptide arrays were blocked using 2% BSA. Master mixes were prepared using the reagents in the PTK and STK kit (PamGene). For the PTK peptide array we used 10 µg protein/array, and for the STK peptide array 5 µg protein/array was used. Images were made using the PamStation 12 platform.

Data was analyzed using the BioNavigator^®^ software (**Supplementary table 1**). The median final score, a combination of the sensitivity score (difference between the THP-1 WT and MKD-MA cells) and the specificity score (active kinase prediction on basis of phosphorylated peptides), was used as ranking factors. The mean kinase statistic indicates the change in kinase activity, with values above zero defined as activation. Predicted kinases with a median final score > 1.2 were visualized on a phylogenetic kinome tree using CORAL (28). For the clustering by functional enrichments, we used the functional annotation clustering tool of the Database for Annotation, Visualization and Integrated Discovery (DAVID) bioinformatics resource version 2021q4 (29, 30), with as background a list of human kinases obtained from KinHub (31). Clusters with an ‘enrichment score’ greater than 1.3 were considered significant.

### Statistical analysis

We used GraphPad Prism 9.1.0 software to create figures and perform statistical analysis, as indicated in the figure legends.

## Results

### Generation and biochemical validation of MK-deficient THP-1 cells

**Figure 1:**
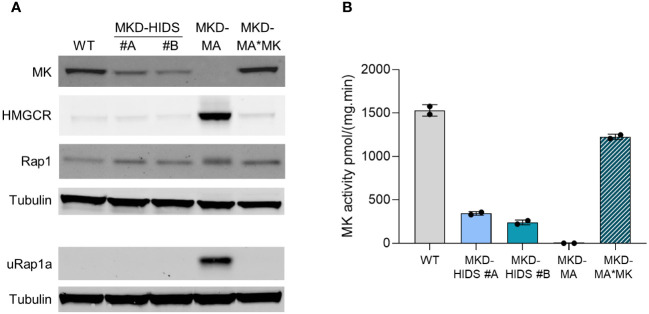
Characterization of WT, MKD-HIDS, MKD-MA and MKD-MA*MK THP-1 cells cultured for 24 hours under standard culture conditions. **(A)** Immunoblot analysis of MK, HMGCR, Rap1 and unprenylated Rap1a (uRap1a). **(B)** MK activity in pmol/(mg.min) indicated as mean ± SD of duplicate measurements.

With the aim to generate immunological relevant cell models for the MKD-MA and MKD-HIDS phenotypes, we targeted the *MVK* gene in the human monocytic THP-1 cell line using the CRISPR/Cas9 genome editing technology. To generate a severe MK deficiency mimicking the MKD-MA phenotype, we first attempted to generate a complete knockout of the *MVK* gene by targeting PAM sites in exon 4 and 5. This turned out to be unsuccessful, most likely because a complete lack of MK does not allow cell survival (32–34). As an alternative, we targeted exon 11, which is the last exon of the *MVK* gene encoding the C-terminus of MK that does not contain the catalytic site but appears important for the stability of the protein. This approach eventually resulted in the identification of one clonal cell line, which was compound heterozygous for a 10 bp and an 18 bp deletion (**Supplementary figure 1**) in exon 11 of *MVK* (MKD-MA THP-1). Similar to cells from MKD-MA patients, the MK activity in this cell line was below detection level with almost no detectable MK protein and high excretion of mevalonic acid into the culture medium (**Figure 1**; **Table 1**). Because we only obtained one clonal MKD-MA cell line, we stably expressed WT *MVK* cDNA in the MKD-MA THP-1 cells (THP-1 MKD-MA*MK) to ascertain that any metabolic, immunological and cellular changes that occur in this MKD-MA cell line are caused by the introduced MK deficiency and not by an off-target event introduced by the CRISPR/Cas9 editing. The expression resulted in similar MK activities and protein levels as observed in WT THP-1 cells and low levels of mevalonic acid secreted into the medium (**Figure 1**; **Table 1**).

**Table 1:** Mevalonic acid measured in supernatants of WT, MKD-HIDS, MKD-MA and MKD-MA*MK THP-1 cells cultured for 24 or 72 hours under standard culture conditions.

THP-1	Mevalonic acid (µM)	
	24 hours	72 hours
WT	ND	ND
MKD-HIDS #A	ND	ND
MKD-HIDS #B	ND	0.10 ± 0.06
MKD-MA	168 ± 5	886 ± 34
MKD-MA*MK	5.7 ± 0.6	23 ± 2

Data is shown as average of an experiment performed in triplo ± SD, or as not detectable (ND).

To generate a milder MK deficiency mimicking the MKD-HIDS phenotype, we aimed to introduce the c.1129G>A (p.V377I) variant in exon 11 of the *MVK* gene of THP-1 using a CRISPR/Cas9 knock-in strategy. The c.1129G>A is the most common variant associated with the MKD-HIDS phenotype (4, 5, 12). After the first round, only a few heterozygous cell clones were obtained, one of which was subsequently used for a second round, which eventually resulted in two clonal cell lines that were homozygous for the variant as confirmed by Sanger sequencing (**Supplementary figure 1**). The relative MK activities in the two cell lines were 22% and 16%, respectively, which corresponds with the protein levels on immunoblots (**Figure 1**) as had been noted previously. Mevalonic acid in the medium of these cells was hardly detectable (**Table 1**).

### Implications of MK deficiency in THP-1 cells for the isoprenoid biosynthesis pathway

To study the impact of the different MK deficiencies on the isoprenoid biosynthesis pathway, we determined the protein levels of HMG-CoA reductase (HMGCR). HMGCR is the rate-limiting enzyme of the pathway, the expression of which is dependent on the levels of isoprenoid end products as mediated by different end-product-feedback regulatory mechanisms (35). The HMGCR level was strongly increased in the MKD-MA THP-1 cells, but in the MKD-HIDS THP-1 cell lines, the levels were similar to the level in the WT cells (**Figure 1A**).

Previously, we and others showed that the MK deficiency in patients’ cells affects the geranylgeranylation of small GTPases (20–24). To study the impact of the MK deficiency on protein geranylgeranylation, we determined the protein levels of the unprenylated form of small GTPase Rap1a (uRap1a). In the MKD-MA THP-1 cell line, uRap1a was markedly increased, whereas it was undetectable in the MKD-HIDS and WT THP-1 cells. The re-expression of WT MK in the MKD-MA THP-1 cell line resulted in MK, HMGCR and uRap1a protein levels that are similar to the corresponding levels in the WT cells (**Figure 1A**).

**Figure 2:**
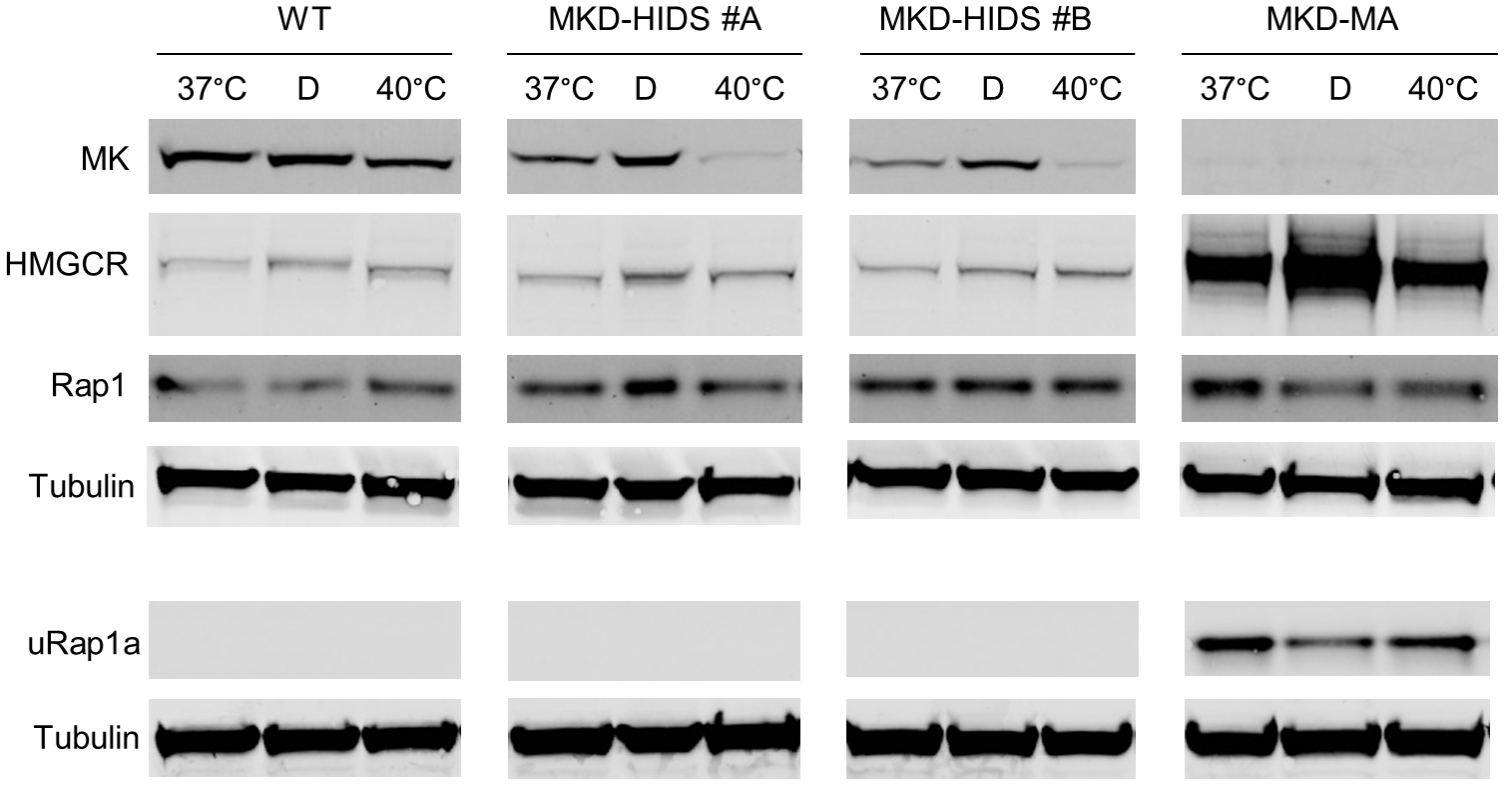
Effect of culturing WT, MKD-HIDS and MKD-MA THP-1 cells for 24 hours in the presence of delipidated FBS or at elevated temperature. Immunoblot analysis of MK, HMGCR, Rap1 and unprenylated Rap1a (uRap1a) of THP-1 cell cultured at the indicated temperatures in standard medium with 10% FBS, or at 37°C in the presence of 10% delipidated FBS (D).

The transcription factor SREBP-2 is activated when cellular cholesterol levels are low, which results in enhanced transcription of genes encoding enzymes of the isoprenoid biosynthesis pathway (36). Accordingly, culturing the WT and MKD-HIDS THP-1 cell lines in lipid (cholesterol)-depleted medium resulted in increased MK and HMGCR protein levels (**Figure 2**). In the MKD-MA THP-1 cells, however, MK protein levels remained barely detectable, while HMGCR protein levels increased even further. This even resulted in a small decrease of uRap1a levels.

Finally, we studied the consequences on the pathway of culturing the THP-1 cell lines at elevated temperature. In fibroblasts of MKD patients, elevated temperature have been shown to cause a further decrease in residual MK activity, which results in a temporary block in isoprenoid biosynthesis as is evident from increased levels of HMGCR (37). Culturing the THP-1 cells at 40°C for 24 hours also decreased MK protein levels in the WT as well as the MKD-HIDS THP-1 cells (**Figure 2**). In the MKD-HIDS THP-1 cells this resulted in very low MK proteins levels. Surprisingly, however, we noted only a small increase in HGMCR levels and no uRap1a was observed, indicating that there was still sufficient flux through the pathway. Even when the MKD-HIDS THP-1 cells were cultured for 3 days at 40°C, no uRap1a was observed (**Supplementary figure 2**). Also mevalonic acid levels in the culture medium showed only a moderate increase (**Supplementary Table 2**). Only when the MKD-HIDS THP-1 cells were cultured in the presence of low levels of simvastatin, there was a clear increase of uRap1a, which was stronger than observed for similarly treated THP-1 WT cells (**Supplementary figure 2**).

### MK-deficient THP-1 cells have a pro-inflammatory phenotype

**Figure 3:**
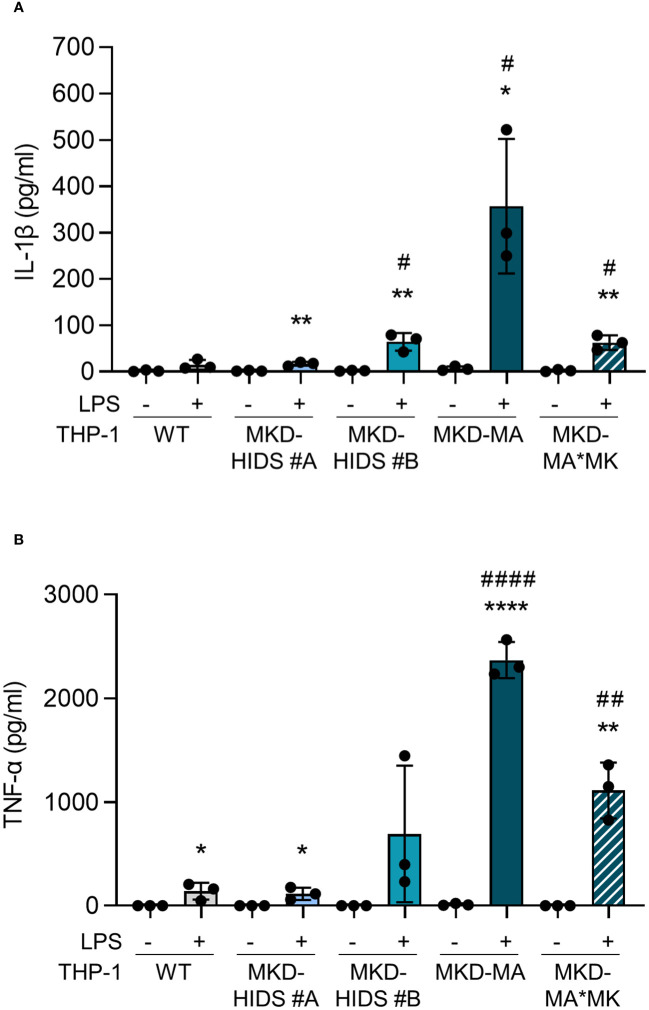
Cytokine release by THP-1 WT, MKD-HIDS, MKD-MA and MKD-MA*MK cells. Cells were cultured for 20 hours followed by an additional 4 hours of culturing with 0 or 10 ng/ml LPS. **(A)** IL-1β release and **(B)** TNF-α release measured in the supernatants. Data are presented as mean ± SD of three independent experiments. Statistical analysis was performed using unpaired t tests to compare unstimulated and LPS-stimulated cytokine release for each cell line, *p-value < 0.05, **p-value < 0.01, ****p-value < 0.0001, and unpaired t tests to compare the LPS-stimulated cytokine release of the MKD-HIDS, MKD-MA and MKD-MA*MK to the WT THP-1 cells, #p-value < 0.05, ##p-value < 0.01, ####p-value < 0.0001.

After the confirmation that the MKD-MA and MKD-HIDS THP-1 cells biochemically behave similar as previously found for MKD patient cells, we studied inflammatory characteristics of the different cell lines upon stimulation for 4 hrs with LPS (**Figure 3**). Previously, it was shown that the LPS-stimulated cytokine release of PBMCs from MKD patients is much stronger than of control PBMCs (14, 18). In line with this, we found that the LPS-stimulated release of IL-1β and TNF-α is markedly increased in the MKD-MA THP-1 cells and moderately in the MKD-HIDS THP-1 cells when compared to the WT cells. The increased cytokine response was reduced in the MKD-MA*MK THP-1 cell line.

**Figure 4:**
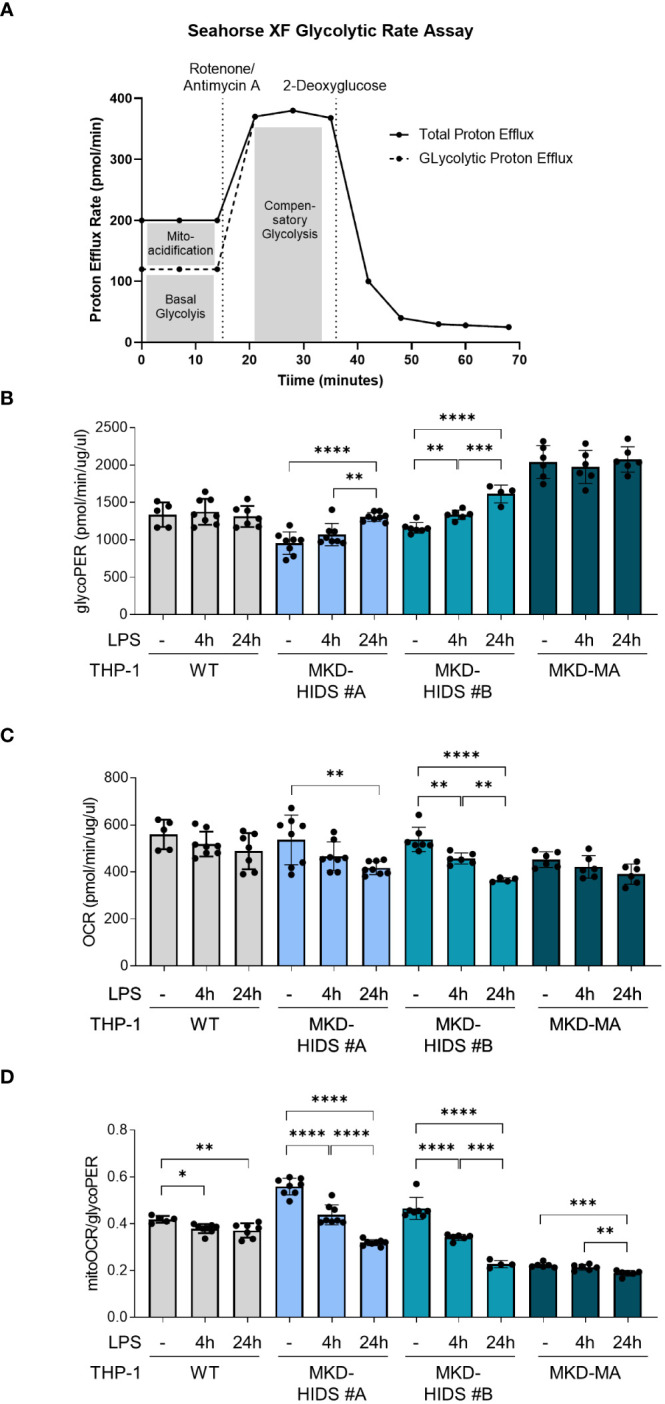
Metabolic reprogramming in MK-deficient THP-1 cells. WT, MKD-HIDS and MKD-MA THP-1 cells were stimulated for 4 or 24 hours with LPS. **(A)** Schematic overview of Glycolytic Rate Assay (adjusted from Agilent, Seahorse XF Glycolytic Rate Assay). **(B)** Basal glycolysis indicated as proton efflux rate (glycoPER). **(C)** Mitochondrial oxygen consumption rate indicated as mitoOCR. **(D)** The ratio mitoOCR over glycoPER. Data are presented as mean ± SD of at least four replicates. Statistical analysis was performed using one-way ANOVA followed by Tukey’s multiple comparison test for each cell line, *p-value < 0.05, **p-value < 0.01, ***p-value < 0.001, ****p-value < 0.0001.

In addition to the increased release of pro-inflammatory cytokines, a key characteristic of pro-inflammatory immune cells is the reprogramming of their metabolism, i.e. a switch from mitochondrial oxidative phosphorylation towards glycolysis, also known as the Warburg effect. The increased glycolysis allows the cells to rapidly produce ATP and produce biomolecules that are crucial for their functioning (38). When we cultured the cells under standard conditions at 37°C, we indeed observed that the basal rate of glycolysis (glycoPER) was significantly increased and the mitochondrial oxygen consumption rate (mitoOCR; a measure for oxidative phosphorylation) decreased in the MKD-MA THP-1 cells when compared to the THP-1 WT cells (**Figure 4**). Moreover, stimulation with LPS for 4 and 24 hours resulted in a significant increase of glycoPER and decrease of mitoOCR in the MKD-HIDS THP-1 cells, but not in the WT and MKD-MA THP-1 cells. This LPS-stimulated metabolic switch in the MKD HIDS THP-1 cells becomes even more clear when expressing mitoOCR and glycoPER as a ratio. The mitoOCR/glycoPER ratio in non-stimulated MKD-MA THP-1 cells was already markedly decreased when compared to the THP-1 WT cells.

### Pharmacological rescue of the MKD-MA THP-1 cellular phenotype by GGOH and TAK-475

**Figure 5:**
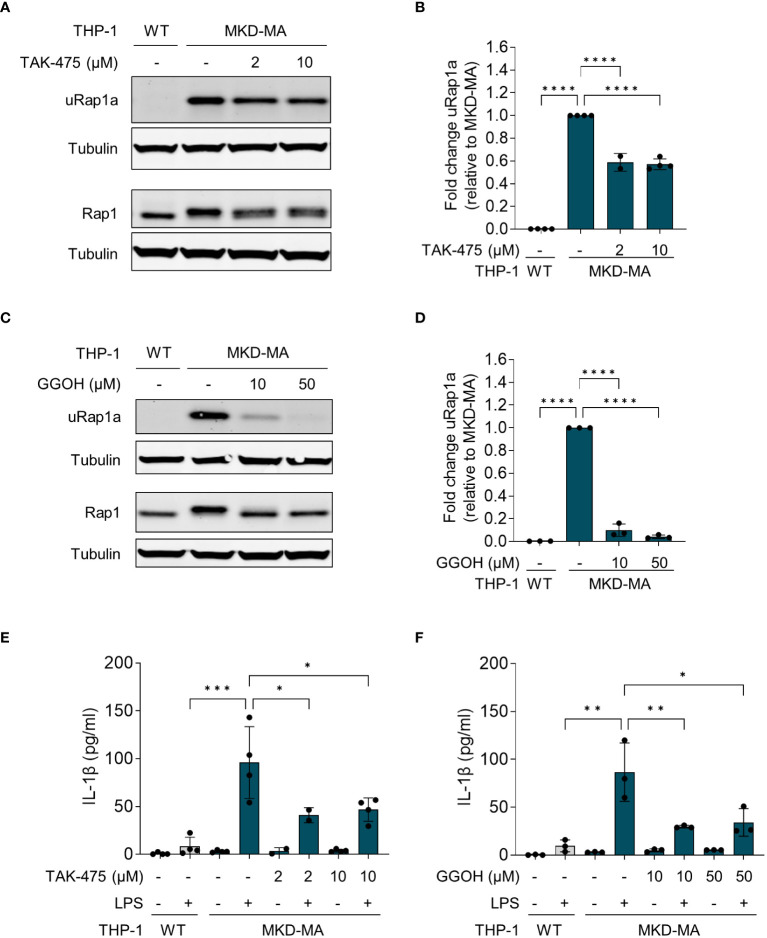
Pharmacological rescue of unprenylated Rap1a (uRap1a) protein levels and IL-1β release. MKD-MA THP-1 cells were cultured for 20 hours in the presence of TAK-475 or GGOH and stimulated for an additional 4 hours with 10 ng/ml LPS. Immunoblot analysis of uRap1a, total Rap1 and tubulin in MKD-MA THP-1 cells cultured in the presence of LPS and TAK-475 **(A)** or GGOH **(C)**. uRap1a protein levels were quantified and expressed relative to the vehicle control treated MKD-MA THP- cells **(B, D)**. IL-1β release was measured in the supernatants of WT and MKD-MA THP-1 cells cultured in the presence of TAK-475 **(E)** or GGOH **(F)**. Data are presented as mean ± SD of at least two independent experiments. Statistical analysis was performed using one-way ANOVA followed by Dunnett’s multiple comparison test, *p-value < 0.05, **p-value < 0.01, ***p-value < 0.001, ****p-value < 0.0001.

The pro-inflammatory phenotype of PBMCs from MKD patients can be reversed by supplementation of GGPP or its precursor geranylgeraniol (GGOH), or by squalene synthase inhibitors (14, 17). Squalene synthase catalyses the first reaction of the sterol isoprenoid-generating branch of the isoprenoid pathway. Blocking this enzyme will re-direct the pathway flux towards the synthesis of GGPP and simultaneously reduce the synthesis of cholesterol. As mentioned above, the reduction in cholesterol levels will activate the SREBP-2-enhanced transcription of the genes encoding enzymes of the isoprenoid biosynthesis pathway. Culturing the MKD-MA THP-1 cells in the presence of the squalene synthase inhibitor TAK-475 or with GGOH also resulted in decreased levels of uRap1a as well as a reduction in the LPS-stimulated release of IL-1β (**Figure 5**).

### Increased protein kinase activity in MKD-MA THP-1 cells

**Figure 6:**
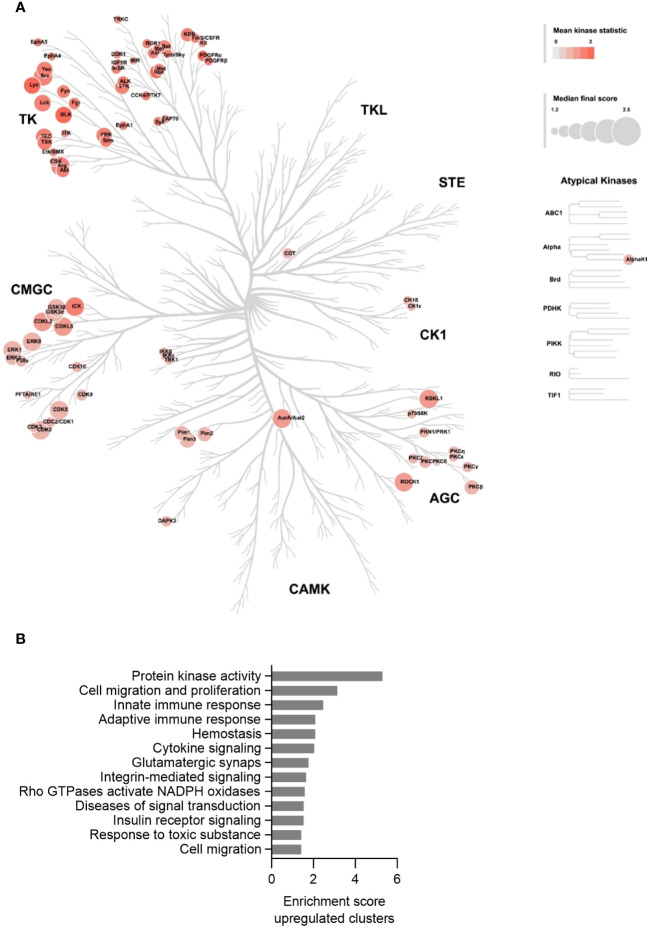
Kinase activity profiling in the MKD-MA THP-1 cell line. **(A)** Mapping of predicated kinases (Median Final Score > 1.2) with increased activity in the MKD-MA THP-1 cell line compared the WT THP-1 cells on a phylogenetic kinome tree. The color of the nodes indicates the kinase statistic. The node size indicates the Median Final Score. The kinome tree was created using CORAL. Data is based on three independent experiments. **(B)** Functional annotation clustering of the predicated activated kinases in the MKD-MA THP-1 cells. For used datasets see **Supplementary table 1**.

After having established that the MKD THP-1 cells biochemically, metabolically and immunologically behave similar as previously reported for MKD patient cells and thus represent a valid cellular model system for MKD, we aimed to identify which signalling pathways are affected in the MKD THP-1 cells. Because small GTPases typically are involved in the regulation of signal transduction pathways that comprise protein kinases, we hypothesized that the compromised geranylgeranylation of GTPases in MKD will have consequences for the activity of such protein kinases. We therefore performed comparative kinome analysis of MKD-MA and WT THP-1 cells using the PamGene functional kinase activity profiling technology. To this end, cell lysates were applied onto PTK (protein tyrosine kinases) or STK (serine/threonine kinases) peptide arrays and the phosphorylation levels of the peptides quantified, based on which the identity and activity of kinases can be predicted. This revealed that the activity of 80 different predicted protein kinases was increased in the MKD-MA THP-1 cells when compared to the THP-1 WT cells. Visualization of these kinases on a phylogenetic kinome tree showed clustering of the protein kinases in multiple subfamilies (**Figure 6A**). This was in particular the case for different subfamilies of the protein tyrosine kinases, including the subfamilies of Src, Syk, Tec, Axl, Met, Ret and InsR kinases of which many or all family members were predicted to be activated. For the serine/threonine protein kinases there was clustering in the Pim and PKC subfamily. In addition, multiple kinases of the CDK, CDKL and MAPK subfamilies were predicted to be activated.

**Figure 7:**
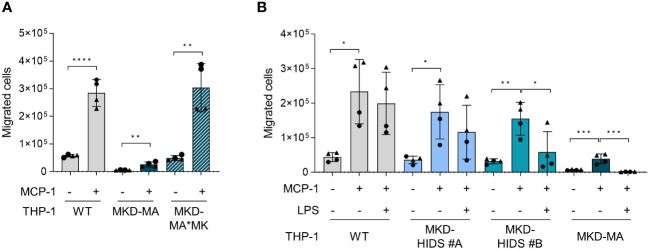
Chemotaxis in WT, MKD-HIDS, MKD-MA and MKD-MA*MK THP-1 cells. **(A)** MCP-1-dependent chemotaxis of WT, MKD-MA and MKD-MA*MK THP-1 cells cultured for 24 hours under standard culture conditions. Significance was determined within each cell line using unpaired t-tests, **p-value < 0.01, ****p-value < 0.0001. **(B)** MCP-1-dependent chemotaxis of WT, MKD-HIDS and MKD-MA THP-1 cells cultured for 24 hours in the presence or absence of 10 ng/ml LPS. Data are presented as mean ± SD of two independent experiments performed in duplicate. Different symbols represent results from independent experiments. Statistical significance was determined for each cell line by one-way ANOVA followed by Dunnett’s multiple comparison test compared to MCP-1 stimulated chemotaxis, *p-value < 0.05, **p-value < 0.01, ***p-value < 0.001.

Functional enrichment analysis of the activated protein kinases identified multiple immune-related processes, including the innate and adaptive immune response and cytokine signalling (**Figure 6B**), supporting the pro-inflammatory phenotype of the MKD-MA cells. One of the most enriched clusters was cell migration and proliferation. In line with this, we found that the MCP-1-dependent chemotaxis in the MKD-MA THP-1 cells was impaired when compared to WT cells, and was restored in the MKD-MA*MK cell line (**Figure 7A**). Interestingly, when we then studied the effect of LPS on chemotaxis, we found that LPS negatively affected chemotaxis in all the cell lines and this effect was stronger in the MKD cell lines than in the WT cells (**Figure 7B**).

**Figure 8:**
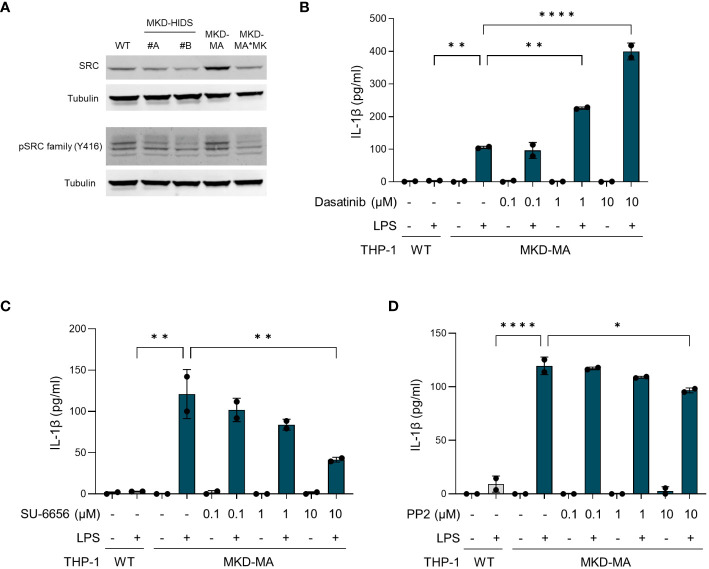
Src kinases in MK-deficient THP-1 cells. **(A)** Immunoblot analysis of Src and Src family kinases in WT, MKD-HIDS, MKD-MA and MKD*MK THP-1 cells. **(B-D)** IL-1β release by MKD-MA THP-1 cells cultured for 1 hour in the presence of the indicated concentrations of **(B)** Dasatinib, **(C)** SU-6656 or **(D)** PP2, after which LPS was added to a final concentration of 10 ng/ml and the cells were cultured for another 4 hours. Data are presented as mean ± SD of two replicates. Statistical analysis was performed using one-way ANOVA followed by Dunnett’s multiple comparison test, *p-value < 0.05, **p-value < 0.01, ***p-value < 0.001, ****p-value < 0.0001.

For further confirmation of the kinome data, we studied the total and phosphorylated levels of tyrosine kinase Src, which is a member of the Src kinase subfamily (**Figure 8A**). This showed that in particular the total levels of Src were increased in the MKD-MA THP-1 cells when compared to the other cell lines. The remarkable observation that incubation of MKD-MA THP-1 cells with the Src kinase-family inhibitor dasatinib resulted in a further increase of the LPS-stimulated IL-1β release (**Figure 8B**), while incubation with the Src kinase-family inhibitors SU-6656 or PP2 resulted in a decrease thereof (**Figures 8C, D**) indicates that different Src kinases may have different roles in the regulation of the inflammatory response.

## Discussion

Many of the currently known biochemical and cellular consequences of the enzyme defect in isoprenoid biosynthesis in MKD have been identified through studies with primary skin fibroblasts or PBMCs from MKD patients. Because inflammatory and immunological consequences cannot be studied properly in skin fibroblasts and PBMCs are difficult to obtain, we introduced MK deficiency in the human THP-1 monocyte cell line using the CRISPR/Cas9 genome editing technology. Functional characterization of the genetically modified MKD THP-1 cells confirmed that they resemble the mild, MKD-HIDS, and severe, MKD-MA, end of the MKD spectrum. In addition to the biochemical and cellular phenotypes established in previous studies with patients’ fibroblasts, the MK-deficient THP-1 cells lines have a pro-inflammatory phenotype, which, similar to patients’ cells (14, 16, 18, 39), is characterized by the enhanced LPS-stimulated release of pro-inflammatory cytokines, including IL-1β.

Biochemically, the MKD-MA THP-1 cell line represents the severe end of the MKD spectrum. MK protein levels are barely detectable and MK activity is below the detection level. The markedly increased HMGCR protein levels and high levels of mevalonic acid in the culture medium when cultured under standard culture conditions indicate that the cells try to compensate for the reduced flux through the isoprenoid biosynthesis pathway. Yet, the presence of uRap1a under this condition indicates that the flux through the pathway is still impaired. In line with previous studies (14, 17), we found that the increased LPS-stimulated IL-1β release in this cell line can be reduced partially by the supplementation of GGOH or by the squalene synthase inhibitor TAK-475, which re-directs the isoprenoid biosynthesis pathway towards the synthesis of non-sterol isoprenoids. This confirms the important role of the non-sterol isoprenoid GGPP in the hyper-inflammatory phenotype of MKD and makes supplementation of intermediate isoprenoids and/or inhibition of squalene synthase interesting options for potential treatment to prevent inflammatory episodes in MKD patients (34).

Compared to the MKD-MA THP-1 cell line, the MKD-HIDS THP- cell lines have higher residual MK protein and activity levels and, accordingly, are less affected with regard to the biochemical and cellular processes studied here. The c.1129G>A (p.(V377I)) variant, which we introduced in the *MVK* gene of these cell lines, is the most common pathogenic variant found in MKD patients and is strongly associated with the milder MKD-HIDS presentation. The variant does not affect the catalytic activity, but impacts correct folding, assembly and/or stability of the mutant enzyme (11, 37). This is reflected by the observation that in general there is a good correlation between the residual MK activity and MK protein levels in cells. Most of the diagnosed MKD patients are compound heterozygous for the p.V377I and a second pathogenic variant (4, 5, 12). A previous study showed that, based on the carrier frequency of the p.V377I pathogenic variant in the Dutch population, patients homozygous for the p.V377I pathogenic variant are under-represented in the group of diagnosed MKD-HIDS patients. This indicates that not all patients who are homozygous for the p.V377I variant develop clinical symptoms (40), which indeed was confirmed in a subsequent study (41). This also explains the mild biochemical, cellular and immunological phenotypes these cells exhibit when compared to the MKD-MA cells even when the cells were cultured for 3 days at elevated temperature, which resulted in depressed MK protein levels, but did not result in increased HMGCR and uRap1a levels. Only when the flux through the isoprenoid biosynthesis pathway is pinched off by low concentrations of the HMGCR inhibitor simvastatin, we observed higher uRap1a protein levels in the MKD-HIDS THP-1 cells when compared to the WT THP-1 cells. This increased sensitivity towards simvastatin inhibition corresponds with previous findings in MKD fibroblasts (20, 21). Taken together, the mild phenotype we observe in our MKD-HIDS THP-1 cells corresponds with the clinical presentation of MKD-HIDS patients at the mildest end of the MKD spectrum.

Metabolic flux analyses of the MK-deficient THP-1 cells showed that LPS stimulation causes increased glycolytic rates and decreased mitochondrial oxygen consumption rates in the MKD-HIDS THP-1 cell lines, but not in the control cells. Interestingly, the MKD-MA THP-1 cell line already showed an increased glycolytic rate when cultured under standard culture conditions and this rate is not further increased by LPS stimulation. Increased glycolytic rates often paralleled by decreased oxidative phosphorylation are also seen in activated pro-inflammatory macrophages and other immune cells (38) and is one of the hallmarks of trained immunity. Trained immunity is the non-specific memory of innate immune cells and is characterized by an enhanced immune response of innate immune cells upon exposure of triggers (42). Recently, mevalonate was found to induce trained immunity, and monocytes from MKD-HIDS patients showed trained immunity characteristics, including the increased expression of glycolytic genes (39).

To gain more insight in the signalling pathways that are affected in MKD, we compared the kinome of THP-1 WT cells with that of MKD-MA THP-1 cells and identified 80 protein kinases with a predicted increased activity in the MKD-MA THP-1 cells. Functional enrichment analysis indicated that these kinases are involved in multiple immune related processes, which is in line with the pro-inflammatory phenotype of the MKD-MA THP-1 cells. Of these, members of the Src tyrosine kinase family are among the first kinases that are activated downstream of immune receptors (43). Indeed, when we studied the expression of Src, which is in the top ranked protein tyrosine kinases, we observed an increase in total protein levels in the MKD-MA THP-1 cell line, but not in the MKD-HIDS and MKD-MA*MK cell lines. These findings may suggest that targeting protein kinases could be a potential therapeutic approach in MKD and other inflammatory diseases. However, the finding that different Src kinase inhibitors can have opposite effects with regards to LPS-stimulated IL-1β release by the MKD-MA THP-1 cells illustrates that a better understanding of the disease mechanisms and the specific role of kinases therein is required before such inhibitors can be applied (44).

Functional enrichment analysis of the activated protein kinases in MKD-MA THP-1 cells also identified cell migration and proliferation as one of the most enriched gene clusters. Functional testing thereof confirmed that the MCP-1 mediated chemotaxis is strongly reduced in MKD-MA THP-1 cells and decreases in V377I/V377I THP-1 cells stimulated with LPS. Chemotaxis is essential in the immune response, for example to guide immune cells to the site of inflammation and to promote interaction between immune cells (45). A disturbed chemotaxis may thus contribute to a dysregulated immune response. Mechanistically, chemotaxis largely depends on reorganization of the cytoskeleton. Small Rho GTPases are key regulators of the reorganization of the actin cytoskeleton and the microtubules (46). It therefore seems likely that a disturbed prenylation of certain small GTPases in MKD cause disturbances in chemotaxis.

Taken together, using the CRISPR/Cas9 technology we generated MKD-HIDS and MKD-MA THP-1 monocytic cells, which display the biochemical, cellular and immunological phenotypes previously established in MKD patients’ cells and thus represent good models for the disease. The cell lines form a valuable source to study underlying disease mechanisms and therapeutic options of this autoinflammatory disorder.

## Data availability statement

The datasets presented in this study can be found in online repositories. The names of the repository/repositories and accession number(s) can be found in the article/**Supplementary Material**.

## Author contributions

FP: Conceptualization, Formal analysis, Investigation, Methodology, Visualization, Writing – original draft. MT: Conceptualization, Formal analysis, Investigation, Methodology, Visualization, Writing – review & editing. RO: Conceptualization, Formal analysis, Methodology, Writing – review & editing. HW: Conceptualization, Formal analysis, Funding acquisition, Supervision, Writing – review & editing.
