# Supplementary material for: Mevalonate kinase-deficient THP-1 cells show a disease-characteristic pro-inflammatory phenotype
Source: Front Immunol. 2024 Mar 14;15:1379220. doi: 10.3389/fimmu.2024.1379220 (PMC10972877; doi:10.3389/fimmu.2024.1379220)
Supplement: Supplementary file 1 [file DataSheet_1.zip › Supplementary Data/Supplementary Figure 1.pdf]

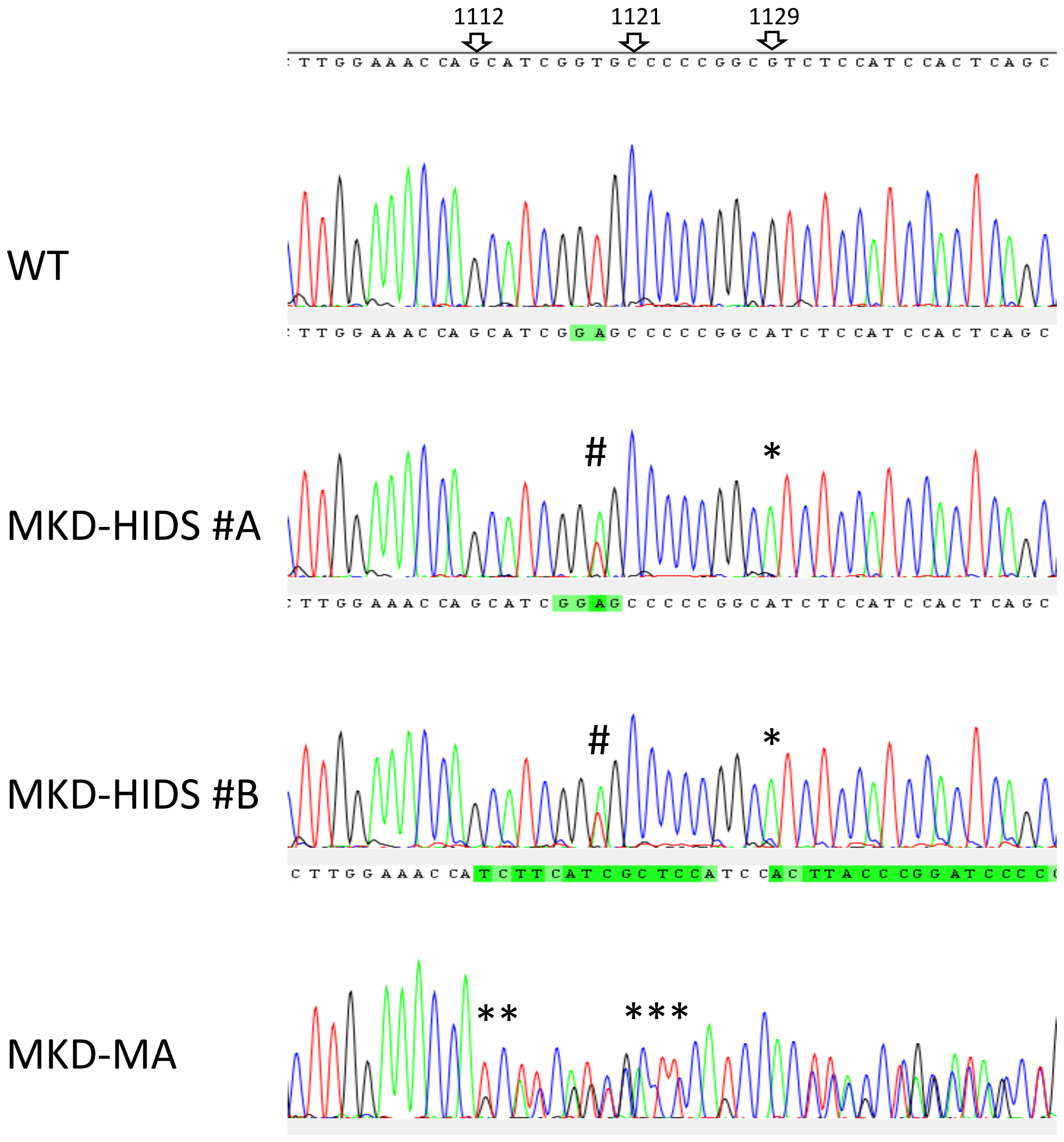

Supplementary figure 1. Chromatograms of exon 11 of the *MVK* gene in THP-1 WT, MKD-HIDS #A, MKD-HIDS #B and MKD-MA cells. #, modified PAM sequence (silent variant), * c.1129G>A (p.(V377I)), ** c.1112_1129del, *** c.1121_1130del.
