# Supplementary material for: Mevalonate kinase-deficient THP-1 cells show a disease-characteristic pro-inflammatory phenotype
Source: Front Immunol. 2024 Mar 14;15:1379220. doi: 10.3389/fimmu.2024.1379220 (PMC10972877; doi:10.3389/fimmu.2024.1379220)
Supplement: Supplementary file 1 [file DataSheet_1.zip › Supplementary Data/Supplementary Figure 2.pdf]

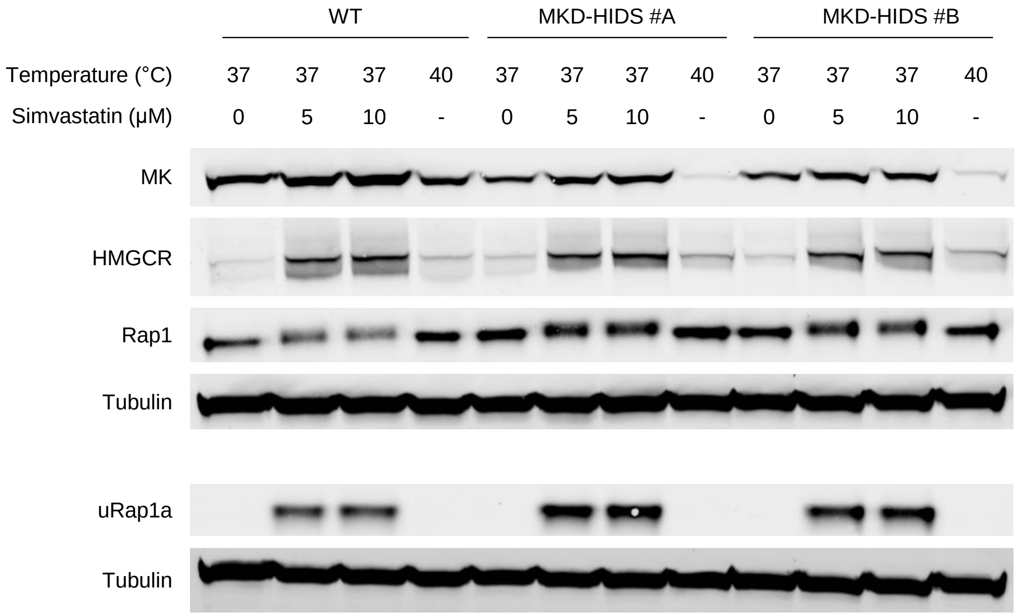

Supplementary figure 2. Immunoblot analysis of MK, HMGCR, Rap1 and unprenylated Rap1a (uRap1a) in WT and MKD-HIDS THP-1 cells. Cells were cultured for 3 days at 37°C in the absence or presence of simvastatin, or for 3 days at 40°C.
