# Supplementary material for: Mevalonate kinase-deficient THP-1 cells show a disease-characteristic pro-inflammatory phenotype
Source: Front Immunol. 2024 Mar 14;15:1379220. doi: 10.3389/fimmu.2024.1379220 (PMC10972877; doi:10.3389/fimmu.2024.1379220)
Supplement: Supplementary file 1 [file DataSheet_1.zip › Supplementary Data/Supplementary Table 2.pptx]

## Slide 1
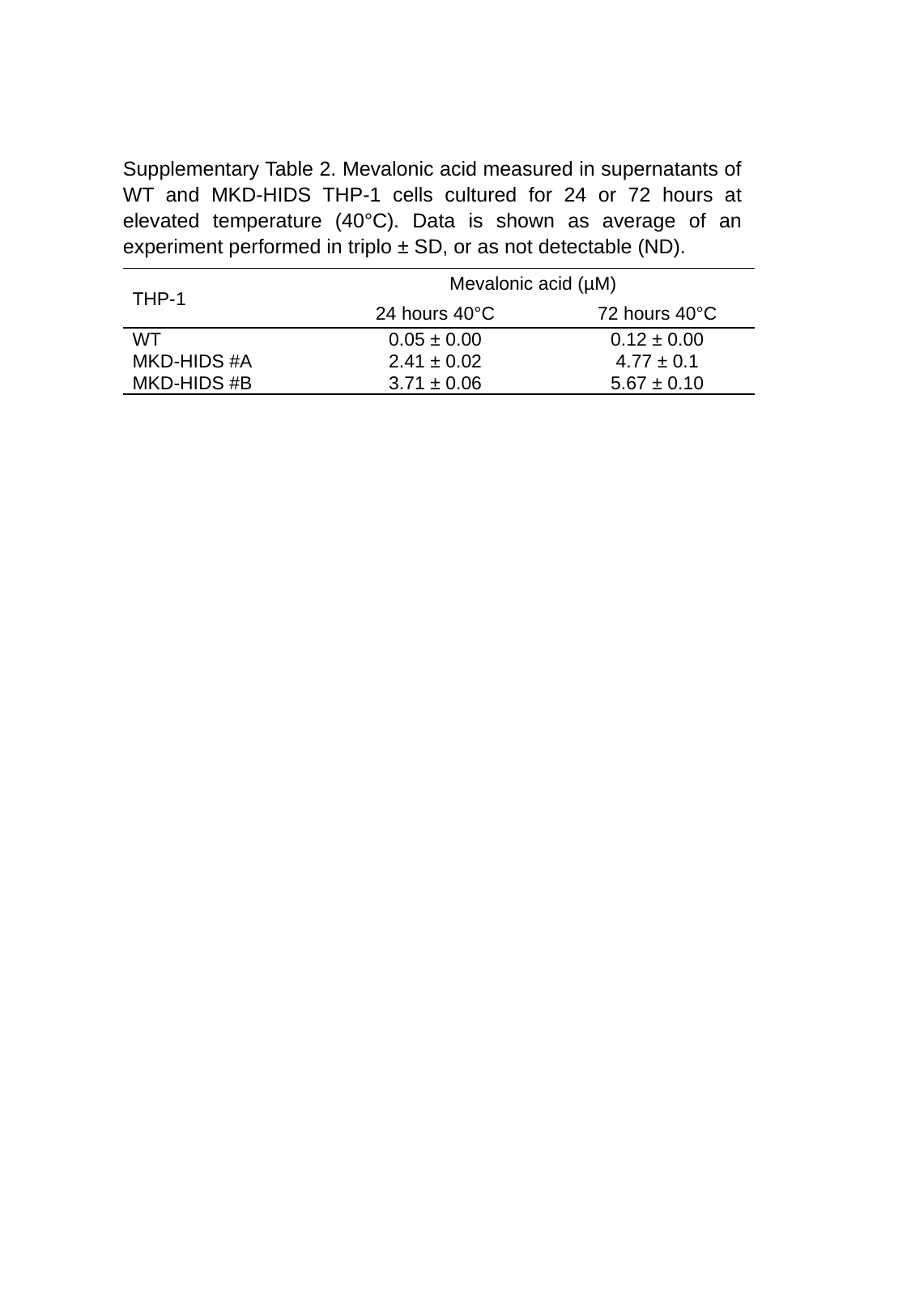

Supplementary Table 2. Mevalonic acid measured in supernatants of WT and MKD-HIDS THP-1 cells cultured for 24 or 72 hours at elevated temperature (40°C). Data is shown as average of an experiment performed in triplo ± SD, or as not detectable (ND).
| THP-1 | Mevalonic acid (µM) | |
| --- | --- | --- |
| | 24 hours 40°C | 72 hours 40°C |
| WT | 0.05 ± 0.00 | 0.12 ± 0.00 |
| MKD-HIDS #A | 2.41 ± 0.02 | 4.77 ± 0.1 |
| MKD-HIDS #B | 3.71 ± 0.06 | 5.67 ± 0.10 |
